# Curvelet Based Offline Analysis of SEM Images

**DOI:** 10.1371/journal.pone.0103942

**Published:** 2014-08-04

**Authors:** Syed Hamad Shirazi, Nuhman ul Haq, Khizar Hayat, Saeeda Naz, Ihsan ul Haque

**Affiliations:** 1 COMSATS Institute of Information Technology, Abbottabad, Pakistan; 2 Centralized Resource Laboratory, University of Peshawar, Peshawar, Pakistan; 3 University of Nizwa, Nizwa, Sultanate of Oman; University of Campinas, Brazil

## Abstract

Manual offline analysis, of a scanning electron microscopy (SEM) image, is a time consuming process and requires continuous human intervention and efforts. This paper presents an image processing based method for automated offline analyses of SEM images. To this end, our strategy relies on a two-stage process, *viz.* texture analysis and quantification. The method involves a preprocessing step, aimed at the noise removal, in order to avoid false edges. For texture analysis, the proposed method employs a state of the art Curvelet transform followed by segmentation through a combination of entropy filtering, thresholding and mathematical morphology (MM). The quantification is carried out by the application of a box-counting algorithm, for fractal dimension (FD) calculations, with the ultimate goal of measuring the parameters, like surface area and perimeter. The perimeter is estimated indirectly by counting the boundary boxes of the filled shapes. The proposed method, when applied to a representative set of SEM images, not only showed better results in image segmentation but also exhibited a good accuracy in the calculation of surface area and perimeter. The proposed method outperforms the well-known Watershed segmentation algorithm.

## Introduction

Electron microscopes (EMs) are the most flexible and powerful instruments available for the analysis and micro-structural characterization of different materials. An ordinary electron microscopy image exhibits high contrast and signal to noise ratio (SNR), with a magnification at millimeter to nanometer scales. Recent advancements in nano-technology have made electron microscopy a very important and powerful tool for the analysis and fabrication of new nano-materials. For a typical EM, the image acquisition time is spanned from several seconds to minutes. Generally, EMs exploit highly energetic beams of electrons in order to examine, on a very fine scale, material objects and their surface features. The measurements of nano-surface features are highly desirable for the characterization of the underlying materials. Scanning electron microscope (SEM), Atomic force microscope (AFM) and X-ray microscope are some of the main instruments used for the characterization of materials at micro- and nano-levels. The problem with surface analysis is that the EM performs live surface analysis of a specimen. This is a costly procedure and should not be carried out on the same sample more than once; offline approaches [1] are therefore recommended for subsequent analyses on the ensued images.

A typical EM operates beyond the visible region of the electromagnetic spectrum. In order to be observed with a naked eye, the output is needed to be mapped on the visible spectrum and recorded as an image. Traditionally, such images had been analyzed by manual scanning wherein the information was required in the form of shapes and sizes of particles, only to be measured by drawing grids across the image. Indeed, this had been a time consuming and laborious exercise that required continuous human intervention and effort. Hence, automated analyses were more than needed and the advent of digital image processing and computer vision techniques did the trick. State of the art digital image processing techniques can be used to acquire the quantitative information from specialized images and such techniques can also be exploited for the enhancement of the EM images.

Image segmentation is mostly used as a pre-processing step to locate objects in SEM images. It is the process of partitioning an image to objects, shapes and regions. To be more elaborate, image segmentation not only focuses on the discrimination between objects and their background but also on the separation between different regions. The image segmentation techniques can be classified as region based and contour based. For region based segmentation [2], [3], morphological operators and watershed approaches are used. The literature is replete with works focused on the segmentation and enhancements of not only the normal images but also the specialized images - especially the images of porous materials, containing voids [4]–[6]. However, the segmentation of an SEM image is a challenging task, especially when it has overlapping objects/regions. These overlapping regions need to be segmented in order to measure their size and shape, without any bias. The watershed algorithm, for its efficiency and ease of use, may be a good choice for segmenting normal images with particular characteristics. The same may not be true in case complex and textured images, like SEM, and the use of watershed segmentation may lead to *over-* or *under-*segmentation.

The proposed method, for automated offline analyses of SEM images, consists of two broad steps:

1. Texture analysis that is achieved by first enhancing the image via the Curvelet transform followed by its segmentation through the application of entropy filter, thresholding and mathematical morphology (MM) operations.
2. A box counting algorithm for estimating the quantities, like surface area and perimeter.

In SEM images, edges are mainly curved in nature and are suited for a Curvelet transform. In addition, the Curvelet transform enables us to perform the analysis with various block sizes. We have applied a Discrete Curvelet Transform to SEM images, in order to get the fine details in the frequency domain.

The rest of the paper is organized as follows. Section surveys the related literature. This followed by Section that presents the proposed method in detail. The ensued results are being analyzed and discussed in Section. The paper is summed up in Section 0.2.

## Related Work

Microscopic images are of vital importance in a wide variety of biological, metallurgical, pharmaceutical, chemical and nutritional materials. The electron microscopy (EM) images are vulnerable to a variety of factors that may degrade the image quality; therefore image enhancement is vital for preserving the quality of information in electron microscope images. A very recent reference [7] in the area of microscope image processing is worth mentioning. Image enhancement is a significant part of image processing, that is used to improve image quality, focus on the interesting features and strengthen the image recognition effects in order to fulfill the demands for special analyses. Its main characteristic is to recover the information contained in an image, for an ordinary or specialized observer. Additionally, it provides better input for other automated techniques used in image processing. Several techniques are adopted for image enhancement. The global image processing techniques - like gradient stretching, gamma correction and histogram equalization - are mainly aimed at improving the underlying image histogram [8]. In contrast, the local processing is better suited for human visual system (HVS) as it focuses on each part of a scene locally. The Retinex [9]–[12] theory of color vision aims to explain how HVS obtain reliable information from the world regardless of variations in illumination. The main clue is that there is little correlation among the extent of radiation dropping on retina and visible lightness of an object.

The main focus of contemporary literature has been the classification on the basis of individual particle characteristics - like size and shape - and their distribution. Texture analysis plays an important and powerful role in microscopic image analysis. In texture analysis, statistical or structural information is produced on the basis of local gray level variations in the image. This information can be used for pattern discrimination. In [13], the authors employ spatial 2D frequency processing tools to analyze the local texture. The work cited in [14], grabs the local spatial variation in image for classification and segmentation. Another example [15] for finding the texture is the use of statistical moments. In [16], the texture analysis is performed for determining the spatial distribution of grains and crystals in minerals. In [17], textural analysis of Synthetic Aperture Radar (SAR) images are performed for the reduction of speckle effects in order to differentiate between forest and non-forest areas. The authors of [18] have used color and surface analysis image processing techniques to distinguish and then quantify wheat ears. For feature extraction, they have relied on texture image segmentation and higher order statistical methods. To overcome the problem of overlaid objects, traditional distance measurement techniques - like K-nearest neighbors and Euclidean distance - have been employed followed by the application of mathematical morphology. The technique in [19] is based on two phases, *viz.* the training phase and the segmentation phase. In the first step, the shape and outer shell knowledge is developed by using the shape histogram and image intensity statistics. The segmentation phase is based on the classical watershed segmentation, k-means clustering and shape alignment stages.

Methodologies, for describing element size and shape, have been formulated in many domains and are used in various geological, chemical, engineering and industrial applications. A wide variety of image processing methods are used in mineral processing, paint and concrete industries [20], [21]. The work, in [22], highlights the potential of such techniques for the quantification of changes in archaeological bones. In [23], segmentation is exploited for observing cortical porosity in bones in 2.5 micron resolution SEM images of the specimen. The method precisely and reproducibly isolates a bone from its background. In addition, it splits the bone into its transitional, cortical and trabecular sections, and perform quantification below and above 100 micron - the size of maximum pores in cortical bone. The authors of [24] carry out legion segmentation of dental images for early carries' detection. The work of [25] employs region adjacency graph for segmentation. Entropy based features are extracted from the texture of script images in [26].

In the context of material science, a range of techniques for three-dimensional (3D) reconstruction of micro-structures had been in vogue for several decades [27]. In the last decade or so, however, there have been a mushroom growth in the field and several methods have been proposed, e.g. [28], thanks mainly to the invention of the focused ion beam microscope and the evolution of transmission electron microscopy into a flexible analytical tool. The main source of the advancement in these fields is perhaps the advent of nano-technology with the aim to achieve nano-scale resolution and the aspiration to get a real 3D view thereof. A texture analysis strategy [29], for nano-fiber membrane, exploits the multivariate statistical techniques and various filters to extract, from the underlying image, the texture features associated with the quality of the material being investigated. In [30], the authors discuss many techniques in order to enhance and segment images of porous materials, obtained through the X-Ray tomography. The study of fractures and their origin is called fractography and one of the first instance, of exploiting the SEM image for this purpose, is found in [31]. A method is proposed in [32] to identify the origin of micron scale cracks based on a genetic algorithm. In [33], a method for quantifying pores in Z-direction is determined and related to optical and physical properties. The authors have used image processing techniques and identified some texture features of powdery samples. One image processing technique [21] provides a layout of system expansion for measuring the micro-structural features of material. In [34], an SEM image analysis tool is used to assay the micro-structure of clay soil and then extract the constituent particles and their boundaries. Using the SEM image analysis, the authors of [20] realize a detailed categorization of sand grains in samples of Thai paddy soils that had been molded from mixed parent materials. In [35], the nature of various fretting particles created by titanium, titanium-molybdenum, and stainless steel are ascertained by employing what the authors call a boundary dilation method. Artificial neural networks had been used to examine the particles; all the three particles produced different results, e.g. titanium showed high diversity of texture and sizes while steel had produced least diversity.

The work in [36] proposes image fusion as solution to compensate for the deficiencies in image enhancement techniques. In a related work [37], low contrast microscopy images were enhanced based on a multi-fusion technique that explores the image edges and details, efficiently, and increases the image contrast and entropy. In [38], two methods of image fusion are proposed. The first one is for exposure fusion in order to reduce the image enhancement deficiencies. The second method is based on the local dissimilarities and maintain the enhanced information. In [39], the authors rely on Sobel operator, Laplacian of Gaussian (LoG) operator and histogram equalization methods for image enhancement in SEM. They propose a multi-technology fusion for low contrast SEM image enhancement wherein the low and high frequency image components are separated to avoid increased image noise.

Techniques, like Wavelet transform filtering and anisotropic diffusion, are useful for edge preservation [30]. They do have limitations, e.g. Wavelets are very powerful technique for 1D signal processing, but may not work well for higher dimensions. In [9], MM and a non-localized algorithm have been employed for noise reduction, image enhancement and object segmentation of multi-layered thin micro-structures. For the detection of breast cancer from microscopic cytology images, a complex Wavelet transform based textural analysis segmentation method is outlined in [40] with a focus on variability features, first and second order texture features, to identify the cancerous cells. Based on a Wavelet framework, line edge detection is carried out in [41] by thresholding the spatial information of top-down nano-scale SEM images. The Wavelet based segmentation can efficiently decompose an image into an approximation image and at least on detailed image. At each level the Wavelet decomposition is performed; the high-frequency components in the image are successively detached. In [42], the authors decompose document images through the Wavelets using the Haar basis function with two levels. The texture features are obtained from the packet decomposition of sub-bands of the Wavelet. They have particular combination of methods results in a significantly reduced over-segmentation.

In [43], [44], the enhancement of ceramic images is performed by using Curvelet and watershed algorithms. They have performed both the enhancement of images and reduction of noise. The Curvelet transform used for image enhancement in [43], [45], is used for representing natural images, sparsely. A Curvelet based approach of image fusion is proposed in [45]. The benefit of Curvelet based technique is that it is receptive to boundaries at various angles and better extracts the high pass or finest details of object contours at multiple scales through multiple nonzero coefficients. A local contrast enhancement method for gray scale images [46] utilizes multi-scale morphological filtering to obtain the scale specific dark and bright features from the input image. In [47], the authors enhance the shape and surface details of an object in a small set of photographs taken from a fixed viewpoint under varying light conditions. They rely on a multi-scale decomposition via a bilateral filter and thereafter reconstruct the image by combining the detail information, at each scale, across all the input images. In [48], the authors realize watershed segmentation using prior shape and appearance knowledge.

## The Proposed Method

In our methodology, the image is first enhanced by obtaining the highest frequency components from its Curvelet transform and then add it to the original image, in order to sharpen the edge detail. Subsequently the sharpened image is subjected to entropy filtering and thresholding to get a binary image, from which boundaries are extracted after morphological processing. In the end, a box-counting algorithm is applied to get the parameters, like surface area and perimeter of the contained objects. We thus propose a novel solution for parameter calculations in electron microscopy that would, in addition, serve as a useful method for the enhancement as well as segmentation of SEM images.

Our procedure involves the following steps:

1. For any segmentation strategy, noise removal is a must, *a priori*, lest one may get a lot of false edges. Our method starts with the removal of unwanted particles or noise present in the image (*I*), through the use of Weiner filter to get *I_W_*. The latter is useful in the situations where the purpose is to reduce noise but preserve the edges. Wiener filter is statistical in nature as it adopts a least square (LS) approach for signal recovery in the presence of noise. It is very effective in eliminating both the additive noise and blur which are usually competing against each other.
2. A Forward Discrete Curvelet Transform (FDCT) is applied to the input image to get the finest detailed coefficients. The FDCT is a multi-dimensional transform in the sense that not only linear contours but also the curvy edges of the contained objects can be captured through its use. Hence, the Curvelet transform captures the structural activity along the radial wedges in the frequency domain and has a very high directional sensitivity. It captures singularities with very few coefficients in a non-adaptive manner. The edge and singularity details are processed to extract the feature points.
3. Next the Inverse Forward Discrete Curvelet Transform (IFDCT) is applied to only the high frequency sub-bands from FDCT domain to get the high pass or detail image (*I_HP_*).
4. The obtained high-pass image (*I_HP_*) is added to *I_W_* and we get an enhanced SEM image (*I_e_*). The image would now have stronger edges than the original and would perform better in lending edge details to the segmentation step.
5. An entropy based filter is thereafter employed to obtain the texture information in the form of (*I_E_*); the texture image highlights the object present in the image. The entropy filter assigns a value to a pixel in the output image according to the entropies of its neighbors in a given range. In our case, the entropy is calculated according to a 9×9 region around the pixel of interest. It is defined by:
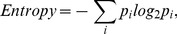
(1)where *p_i_* is the probability that the entropy difference between the pixel of interest and the *i*th neighbor agrees to certain threshold.
6. The entropy filtered image is subsequently thresholded to get a binary image with unwanted objects eventually removed. The threshold ((*T*)) can be intelligently adjusted to restrict the objects according to the size requirement of permitted objects. A Mask is thus generated that has the same size as original image. The mask is used to get the boundaries of objects in an image. This mask would be used to extract only the object(s) of interest, on the fly.
7. The mask is further refined via Mathematical Morphology (MM) processing, getting (*I_M_*), in order to further highlight the image boundaries. The segmented image (*I_S_*) is formed by superimposing the mask (*I_M_*) on the image *I_E_* and the regions are separated by setting all the pixels to 1 that belong to the set of the segmentation boundaries.
8. In the end, a box counting algorithm is applied to the segmented image. Various algorithms are used for calculating the fractal dimensions - like the fractional (or fractal) Brownian motion and triangular-prism-surface area methods. The box counting algorithm counts the number of boxes having side length *r* needed to cover the surface of fractal objects and the number of boxes *N*, occupied by more than one pixel of the image. Two procedures are defined by two parameters in the box counting method. One is the selection of *r* and the other is the other is the range of *r*. The SEM image has finite set of points and the upper limit is the size of image while the lower is the pixel unit. Various researches [42] proposes using 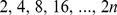 pixels as box sizes to have a uniform spread of observation. The quadratic boxes cover the object, and the number of the boxes is recorded. The fractal dimension (FD) measures the dependence between the number of boxes *N* and the box side length *r* and is defined by

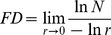
(2)For estimation, the dependence of 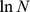 on In *r* is fitted by a line 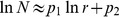
(3)
  Substituting Eq. 3 in to Eq. 2 we get 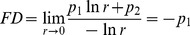
(4)
  The term 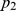 disappears because the denominator tends to infinity. In [49], the authors have considered the FD relationships of images of fracture surfaces. The relationships did not reach the tightness, which would be sufficient for fractographic functions. It was found that images cannot be characterized by a single numerical parameter; the reason being the too multi-formic, complex and uneven character of the fractured surfaces.

The essence of our strategy is outlined in the form of algorithms outlined in Table 1 and Table 2. The former is concerned with the enhancement step - from 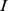 to 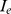 - whereas the latter outlines the main algorithm that requires 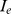 and process it for quantification.

**Table 1:** The Image Enhancement Algorithm.

**Require:**	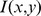
**Ensure:**	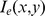 /*Enhanced image*/
1:	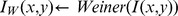
2:	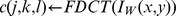
3:	**for** 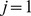 to 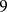 **do**
4:	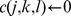
5:	**end for**
6:	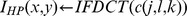
7:	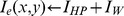
8:	Exit

**Table 2:** The Main Algorithm.

**Require:**	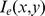 of size 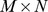
**Ensure:**	The Surface Area ( 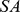 ) and Perimeter ( 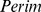 )
1:	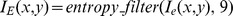 {Subject to 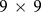 entropy filter}
2:	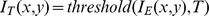 {Threshold the image using threshold 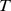 }
3:	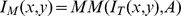 {MM processing to include regions conforming to threshold 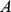 }
4:	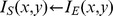
	{lines 5–11: Apply the mask 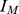 to 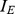 to get the segmented image 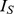 }
5:	**for** 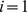 to 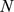 **do**
6:	**for** 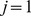 to 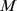 **do**
7:	**if** 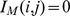 **then**
8:	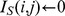
9:	**end if**
10:	**end for**
11:	**end for**
12:	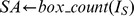
13:	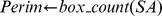
14:	Exit

## Simulation Results

Our method employs a Curvelet transform for the image enhancement and a box counting algorithm for quantification. To demonstrate the efficacy of our method, we have used a data set of 60 SEM images of different materials and at various intensities. In this section we first apply our method to a representative example in detail and then present the results with respect to all the 60 samples.

### 0.1. Example Illustration

**Figure 1:**
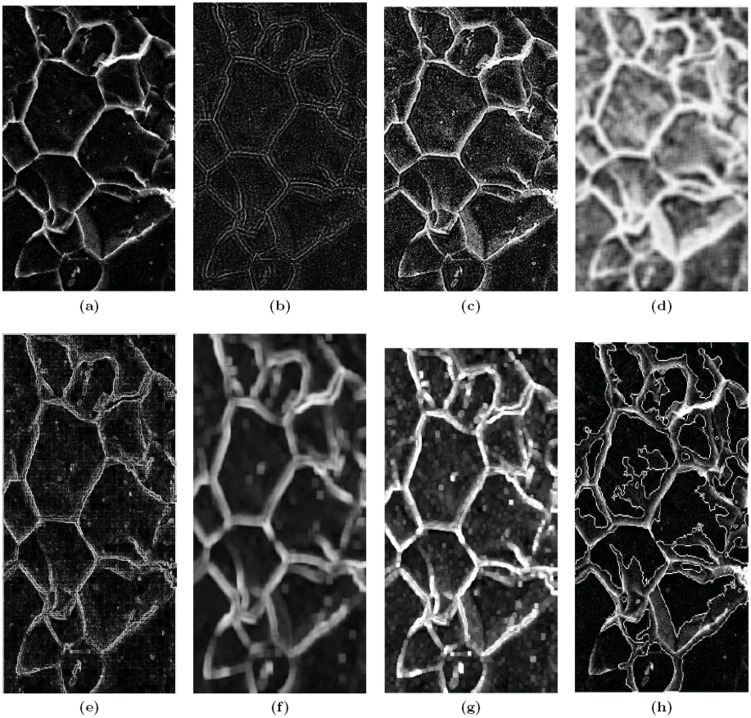
Proposed Method Illustrated. (a) Input Image, (b) Detailed Image, (c) Enhanced Image, (d) Texture Image after Entropy filtering, (e) Corresponding Binary Image, (f) Mask generated from Binary Image, (g) Image generated by Range filter, and (h) Image Segmented by the Proposed Method.

The images shown in Figure 1 are concerned with the various stages of our method as applied to an example image shown in Figure 1.a that corresponds to an SEM image of low carbon steel. This image was subjected to Wiener filtering and FDCT. By setting the lower frequency coefficients to zero and applying the IFDCT, a high-pass detail image (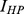) is obtained which is given in Figure 1.b. The high pass image contains the finest details of the image, and we add the high pass image with the smoothened image, we got 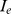, the edge-enhanced image of Figure 1.c. In other words, this is the output image of Algorithm 1 and input to Algorithm 2.

The edge-enhanced image was then subjected to a 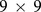 entropy filter for the texture analysis with the aim of region characterization. The textured image is shown in Figure 1.d. The texture of an image provides the required data about the local differences in intensity level of pixels in an image. Next a binary image (Figure 1.e) was realized through the thresholding of the texture image followed by the use of morphological opening in order to remove the small unwanted spots/regions disagreeing with a certain threshold. The result was a mask shown in Figure 1.f which was employed to highlight the region boundaries. For this particular case we had employed a range filter as part of the MM processing to output Figure 1.g. The resultant mask (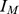) was finally superimposed on 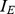 resulting in the segmented image 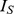. The segmented image is shown in Figure 1.h.

By using a box counting algorithm, quantities - like surface area and perimeter - from different segmented regions in the image were calculated and preserved for further analyses. The perimeter, for example, of a given segmented area was found by using the polygon method and by counting the boundary ‘boxes’, while presenting the results in pixel units.

### 0.2 Detailed results and comparison analysis

The qualitative visual assessment shows that the segmentation results of our proposed algorithm are comparable to those proposed in the literature. We have performed the segmentation of SEM images and calculated the required parameters of different regions in the image. The results are calculated from images of different materials. We have analyzed the image of 14 materials, including low carbon steel, aluminum, copper, carbonate, RBC (red blood cells), pollen grains, clay and iron oxide. We have compared our results with the well-known Watershed segmentation technique. We have estimated the measurement parameters with the both techniques, i.e. our proposed technique and with Watershed segmentation technique [22]. The obtained results have also been compared with the manually calculated results.

**Figure 2:**
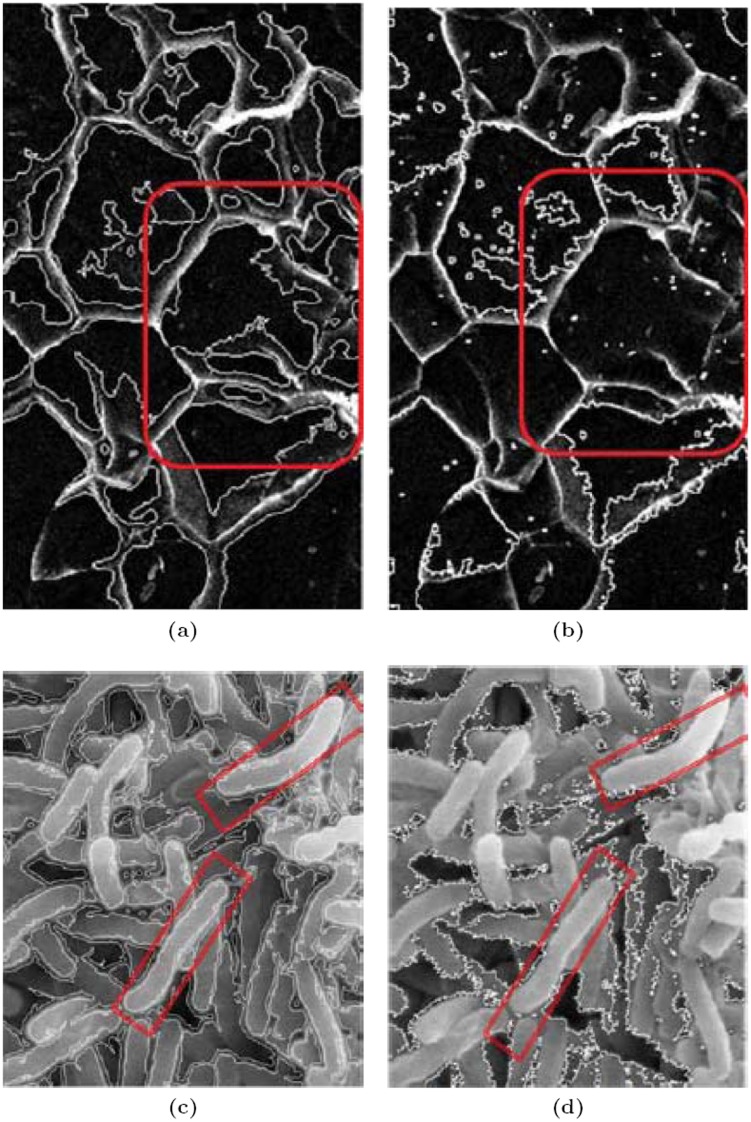
The Proposed Method vs. Watershed Segmentation. (a) Example 1 Segmented by the Proposed Method, (b) Example 1 Segmented by Watersheds, (c) Example 2 Segmented by the Proposed Method, and (d) Example 2 Segmented by Watersheds.

The proposed technique has produced better results than the Watershed segmentation approach. The segmentation results for two different cases are illustrated in Figure 2. The first case is concerned with the segmentation of a SEM image of a Low carbon steel. Figure 2.a shows the resultant of our technique while Figure 2.b that of Watershed segmentation technique. The problem with the Watershed segmentation result is that it suffers from under segmentation for some regions. This problem can be tackled by region merging but the issue with region merging is that how to determine similarity among the different segmented regions. This task becomes more challenging when the images contain dissimilar and complex objects. It performs poorly when there are high textured objects in the image. Obviously, quantifications with our method had been more reliable due to better segmentation. The main drawbacks we have analyzed in different proposed methods in literature for microscopy image quantification and enhancement is that most of the techniques are unable to identify overlapped and connected particles. When overlapping particles appear in different regions of image the existing techniques consider it as single particle, which usually leads to an erroneous characterization of the particles.

**Figure 3:**
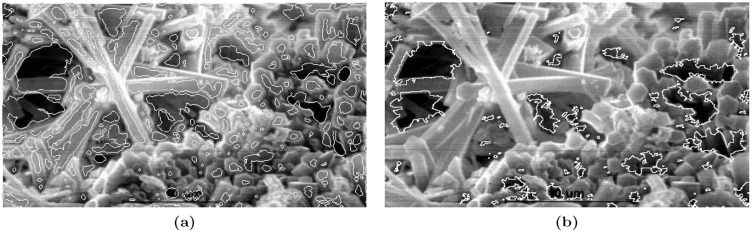
Segmentation of the Carbonates Image. (a) The Proposed Method, (b) The Watershed Method.

Not only under-segmentation but also the tendency to over-segment is also common with the watershed techniques. For illustration compare our result from Figure 2.c with that in Figure 2.d. Another proof of the inability of watershed techniques, to segment properly, in the presence of overlapped particles, is obvious from the carbonate SEM image of Figure 3. For microscopy images, the segmentation method should be able to accurately separate the particles pixels from the background pixels. Our proposed method provides the better solution for image segmentation. The method precisely segment the overlapped particles shown in Figure 3.a as against its watershed counterpart (Figure 3.b). From the results shown in the Figure 2 and Figure 3, we can say that our technique works well in rich textured SEM images.

**Figure 4:**
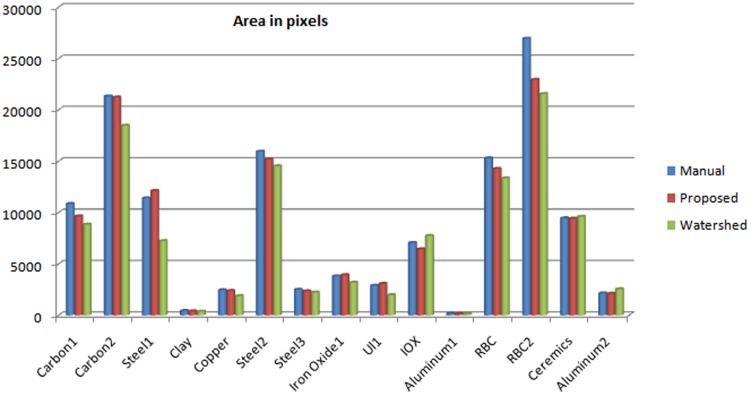
Graph Illustrating the Area Comparison.

Table 3 compares the area of the regions in 15 example SEM images calculated manually with that estimated by the proposed method and watershed segmentation technique. Obviously, the manual results must be the gauge since they are carefully calculated with high precision. It can be readily observed that the proposed method outperforms watershed segmentation in closeness to the reference manual method and the percentage error of the proposed method is very low *par rapport* the Watershed segmentation. Figure 4 graphically illustrates this comparison. The average relative error by the proposed method was observed to be only 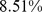, which is very low as compared to that by Watershed segmentation, i.e. 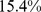.

**Table 3:** Estimated Areas from Segmented SEM Image.

Image Tag	Manual Methods	Proposed Method	Watershed Segmentation
Carbon1	10864	9647	8853
Carbon2	21325	21227	18469
Steel1	11411	12123	7240
Clay	438	402	373
Copper	2464	2394	1890
Steel2	15947	15191	14528
Steel3	2510	2360	2232
Iron Oxide	3801	3929	3193
U1i	2893	3277	1980
IOX	7062	6444	7753
Aluminum1	160	151	146
RBC2	15303	14277	13352
RBC	26943	22943	21556
Ceramics	9468	9430	9610
Aluminum2	2151	2118	2557

The Sample Images Referred in the Tables: Figure 6 lists the sample images referred in Table 3.

**Figure 5:**
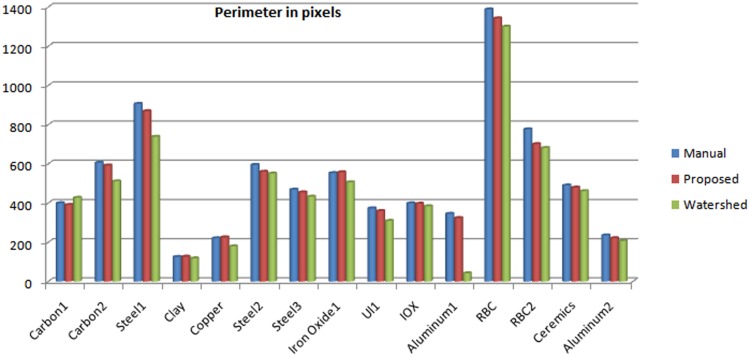
Graph Illustrating the Perimeter Comparison.

In a similar fashion, perimeter values can be compared. First the length of the textural region was ascertained and then the perimeters were derived. The perimeter is inclined to be, roughly, more than double the length, due to inherent porosity that may increase the population of perimeter pixels. In Table 4 we are showing the perimeter of the segmented regions from each of the 15 SEM images, estimated manually and via the proposed and watershed methods. These results are graphically illustrated in Figure 5. In this case too, the proposed method has produced results more closer to the manual ones computed by the means of photometry. The aggregate error rate of proposed method is 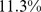 which is far better than the results generated by using Watershed segmentation at 23.5%.

**Table 4:** Estimated Perimeters from Segmented SEM Images.

Image Tag	Manual	Proposed	Watershed
Carbon1	399	391	427
Carbon2	606	592	511
Steel1	906	869	728
Clay	125	127	118
Copper	220	225	180
Steel2	595	561	550
Steel3	469	454	433
Iron Oxide	553	557	506
U1i	373	359	309
IOX	399	393	384
Aluminum1	345	323	242
RBC2	1388	1342	1300
RBC	776	701	681
Ceramics	490	481	460
Aluminum2	235	221	201

**Figure 6:**
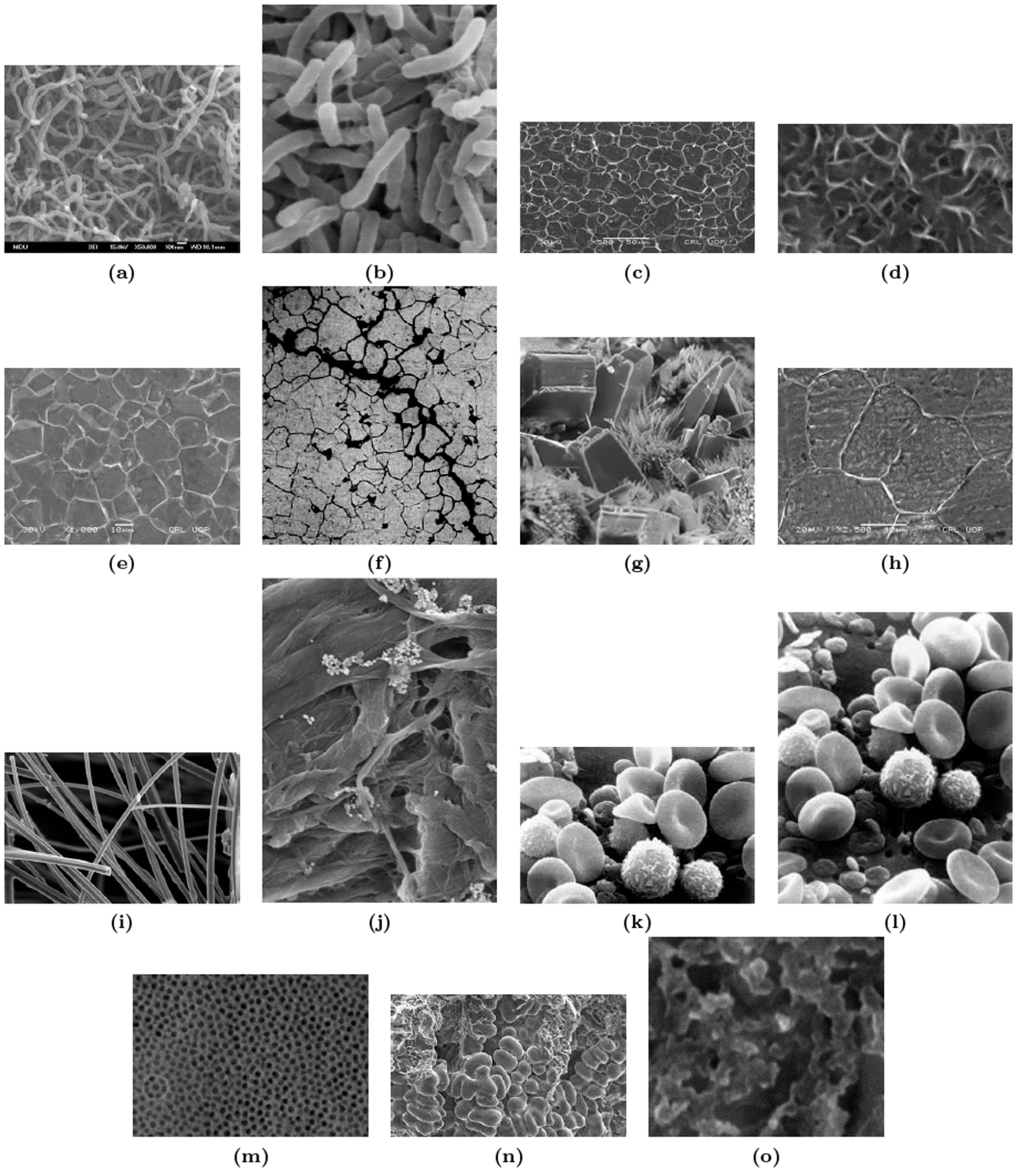
Set of images. (a) Carbon1, (b) Carbon2, (c) Steel1, (d) Steel2, (e) Steel3, (f) Clay, (g) Copper, (h) Iron Oxide, (i) U1i, (j) IOX, (k) RBC, (l) RBC2, (m) Aluminum1, (n) Aluminum2, and (o) Ceramics.

The Sample Images Referred in the Tables: Figure 6 lists the sample images referred in Table 4.

## Conclusion

In this paper, we proposed a novel method for SEM image segmentation and subsequent quantification of the geometrical parameters. Our method is based on two main stages: one is the image segmentation and the other is concerned with the estimation of the underlying measures. The former constitutes a more complex task than the later one, because SEM images usually contain irregular and complex objects. The segmentation part is based on a Curvelet transform and entropy calculations. The Curvelet transform has proved to be very much useful for the multi-dimensional analysis that dealt with not only linear structures but also the curved contours. The technique is promising and may well produce better results than the state of the art techniques, like watershed segmentation. To test its efficacy, we compared our results with those by Watershed segmentation, and found out that the proposed technique has segmented the highly textured SEM images far better than its Watershed counterpart. In future, we want to expand our analyses to nano-scale images while vying for an improved segmentation and measurement strategy.
